# Effect of Complete Replacement of Dry-Rolled Corn with Unprocessed Rye on Growth Performance, Efficiency of Dietary Net Energy Use, and Carcass Traits of Finishing Heifers

**DOI:** 10.3390/ani11010099

**Published:** 2021-01-06

**Authors:** Elizabeth M. Buckhaus, Warren C. Rusche, Zachary K. Smith

**Affiliations:** Department of Animal Science, South Dakota State University, Brookings, SD 57007, USA; Elizabeth.Buckhaus@sdstate.edu (E.M.B.); Warren.Rusche@sdstate.edu (W.C.R.)

**Keywords:** feedlot, grain processing, growth performance, integrated crop–livestock system

## Abstract

**Simple Summary:**

Crop rotation diversity has many benefits for integrated crop–livestock production systems. Cereal rye deserves consideration for use as a component of an integrated crop–livestock system as rye is a multi-use crop than can be grazed, harvested for forage, or harvested for grain and straw. Previous research from this research center has indicated that processed rye (processed to a processing index of 78.8%) is a suitable ingredient (84% of the net energy value of dry-rolled corn) for use in cattle-finishing diets. Processing grains prior to feeding is proven to enhance growth performance in feedlot cattle. A major impediment to the use of rye in finishing diets is that the rye should be processed prior to feeding, and processing rye requires different equipment settings than for processing dry-rolled corn. The objectives of the present study were to evaluate the impact of the complete substitution of dry-rolled corn with unprocessed rye in finishing diets fed to feedlot heifers on growth performance, dietary net energy use, and carcass traits.

**Abstract:**

Continental crossbred beef heifers were used in a randomized complete block design experiment to evaluate the effects of replacement of dry-rolled corn with unprocessed rye on the finishing-phase growth performance and efficiency of dietary net energy (NE) use. Fifty-six heifers (433 ± 34.0 kg) were transported 241 km from a sale barn in North Central South Dakota to the Ruminant Nutrition Center in Brookings, SD. Heifers were blocked by weight grouping and allotted to treatment pens (*n* = 7 heifers/pen and 4 pens/treatment). Treatments included a finishing diet that contained 60% grain (diet dry matter basis) as dry-rolled corn (DRC) or unprocessed rye grain (RYE). On study day 14, all heifers were consuming the final diet and were implanted with 200 mg of trenbolone acetate and 28 mg of estradiol benzoate (Synovex-Plus, Zoetis, Parsippany, NJ, USA). The RYE heifers had decreased (*p* ≤ 0.01) final body weight, average daily gain, and gain efficiency; however, they tended (*p* = 0.08) to have a greater dry matter intake compared to DRC heifers. RYE heifers had decreased (*p* ≤ 0.01) observed dietary NE and decreased (*p* ≤ 0.01) observed-to-expected dietary NE ratio for maintenance and gain compared to DRC heifers. The dressing percentage, 12th rib fat thickness, ribeye area, and the distribution of yield and quality grades were not altered (*p* ≥ 0.12) by dietary treatment. The hot carcass weight, calculated yield grade, estimated empty body fat (EBF), and body weight at 28% EBF decreased (*p* ≤ 0.02) and retail yield increased (*p* = 0.01) in RYE compared to DRC heifers. These data indicate that unprocessed rye is a palatable feed ingredient for inclusion in finishing diets for beef cattle and that rye inclusion only minimally influences the carcass quality grade. The feeding value of unprocessed rye is considerably less (21.4%) than that of dry-rolled corn using current standards and approximately 91% of the NE value of processed rye (processing index = 78.8%). Rye grain fed as processed or unprocessed grain has an NE value that is less than 90% of that of dry-rolled corn.

## 1. Introduction

Crop-rotation diversity has many benefits for integrated crop–livestock production systems. These include yield resiliency and crop yield increases compared to single- or two-crop rotation systems [1]. When combined with livestock production, diversified crop rotation can reduce year-round variation in labor compared to traditional two-crop rotation coupled with livestock production [2].

Cereal rye can be used as a component of an integrated crop–livestock system. Rye is a multi-use crop than can be grazed, harvested for forage, or harvested for grain and as straw. In addition, rye is harvested earlier in the season compared to other traditionally used row crops, allowing for greater flexibility related to manure management or use of shorter-growing-season forage crops to be fed to livestock if weather and market conditions are appropriate. Hybrid rye germplasms, which have recently become available to the United States from Europe, are of particular interest because of a greater yield potential and lesser ergot incidence compared to open-pollinated rye varieties [3].

Previous research from this research center indicated that processed rye (processing index of 78.8%) is a suitable ingredient (84% of the net energy value of dry-rolled corn) for use in finishing diets [4]. Rusche, Walker, Sexton, Brattain, and Smith [4] demonstrated that the apparent net energy (NE) value for the gain in processed rye increases by 12.8% when blended with dry-rolled corn (one-third processed rye and two-thirds dry-rolled corn) in finishing diets fed to yearling feedlot steers compared to complete replacement of dry-rolled corn with processed rye. A major impediment to the use of rye in finishing diets is that rye should be processed prior to feeding. Processing rye requires different equipment or altered settings compared to processing corn as dry-rolled corn; hence, for an operation to use and feed processed rye either a substantial investment or increased feed mill operational complexity is required.

The objective of this experiment was to determine the effects of complete replacement of dry-rolled corn with unprocessed rye on intake, cattle growth performance, and feed conversion efficiency in finishing heifers. Our hypothesis was that unprocessed cereal rye has a lower NE value compared to dry-rolled corn and that rye can be substituted for dry-rolled corn in finishing diets, but would result in poorer growth performance and feed efficiency with no negative effects on carcass characteristics.

## 2. Materials and Methods

The animal care and handling procedures used in this study were approved by the South Dakota State University Animal Care and Use Committee (Approval Number: 2007-031E).

### 2.1. Animal Management and Dietary Treatments

Heifers were used to evaluate the effect of replacement of dry-rolled corn with unprocessed rye on finishing-phase growth performance and the efficiency of dietary NE use. Fifty-six crossbred beef heifers (433 ± 34.0 kg) were transported 241 km from a sale barn in North Central South Dakota to the Ruminant Nutrition Center (RNC) in Brookings, SD, on 24 August 2020. Upon arrival to the RNC, the heifers were housed in 7.62 m × 7.62 m concrete surface pens with 7.62 m of linear bunk space and provided ad libitum access to long-stem grass hay, and water. The following day heifers were offered a common 60% concentrate diet at 2% of body weight. On 27 August 2020 (3 day following arrival), all heifers were individually weighed (scale readability 0.454 kg), given a unique identification ear tag, vaccinated for viral respiratory pathogens (IBR, BVD 1 and 2, PI_3_, and BRSV (Bovi-Shield Gold 5, Zoetis, Parsippany, NJ, USA)) and clostridials (Ultrabac 7/Somubac, Zoetis), and administered pour-on moxidectin (Cydectin, Bayer, Shawnee Mission, KS, USA) according to label directions. On 1 September 2020 (9 day following arrival), all heifers were again individually weighed, and this body weight was used for allotment. The heifers were blocked by weight grouping and allotted to their study pens the following day (*n* = 7 heifers/pen and 4 pens/treatment), and test diets were initiated. Treatments included a finishing diet that contained either (1) dry-rolled corn (DRC) or (2) unprocessed hybrid rye (RYE) as the grain component. On study day 14, all heifers were consuming the final diet and were implanted with 200 mg of trenbolone acetate and 28 mg of estradiol benzoate (Synovex-Plus, Zoetis); an implant retention check was performed on day 42. The initial body weight (BW) was the BW captured on September 2, 2020. Following study initiation, the heifers were transitioned to a high-concentrate diet over the course of 14 d (Table 1). The diets were fortified to provide vitamins and minerals to meet or exceed nutrient requirements, providing monensin sodium at 32.3 g/Mg (DM basis) and melengestrol acetate (MGA, Zoetis) at a rate sufficient to provide 0.50 mg/heifer·d^−1^ [5]. There was no morbidity or mortality noted in the present study. Fresh feed was manufactured twice daily in a stationary mixer (2.35 m^3^; scale readability 0.454 kg) and offered to heifers in equal amounts at each feeding. Orts were collected, weighed, and dried in a forced-air oven at 100 °C for 24 h to determine the DM content if carryover feed went out of condition or was present on weigh days. If carryover feed was present on weigh days, the residual feed was removed prior to BW measurements. The dry matter intake (DMI) of each pen was adjusted to reflect the total dry matter (DM) delivered to each pen after subtracting the quantity of dry orts for each interim period. The actual diet formulation was based upon weekly DM analyses (drying at 60 °C until no weight change) and corresponding feed-batching records. After weekly DM determination (method no. 935.29 [6]), monthly composite samples from each ingredient were analyzed for N (method no. 968.06 [7]; Rapid Max N Exceed; Elementar; Mt. Laurel, NJ, USA) and ash (method no. 942.05 [6]). Corn co-products were analyzed for ether extract content using an Ankom Fat Extractor (XT10; Ankom Technology, Macedon, NY, USA). Percentages of acid detergent fiber (ADF) and neutral detergent fiber (NDF) were assumed to be 3% and 9% for corn and 9% and 19% for rye, respectively. Analysis of the ADF and NDF composition for all other feed diets was conducted as described by [8]. Data presented in Table 1 are actual DM diet composition, monthly composite nutrient concentrations, and tabular energy values [9].

### 2.2. Growth Performance Calculations

The heifers were individually weighed on days −1, 1, 14, 42, and 77. Cumulative growth performance was based upon the shrunk BW from day 1 (4% shrinking applied to account for digestive tract fill) and carcass-adjusted final BW (FBW) from hot carcass weight (HCW) calculated as: HCW/0.625. The energetic assessment period was from day 14 to 77 using the BW from day 14, shrunk by 4%, and the FBW. The average daily gain (ADG) was calculated as the difference in the BW for the period of interest, divided by the days in that period, and feed efficiency was calculated as ADG/DMI.

### 2.3. Carcass Trait Determination

The heifers were harvested when they were visually appraised to have 1.27 cm of rib fat (RF). They were shipped the afternoon following the final BW determination and harvested the next day at Tyson Fresh Meats in Dakota City, NE, USA. The heifers were co-mingled at the time of study termination and remained as such until 7:00 a.m. the morning after shipping. The HCW was captured immediately following the harvest procedure. Video image data were obtained from the plant for the ribeye area, RF, and United States Department of Agriculture (USDA) marbling score. The yield grade was calculated according to the USDA regression equation [10]. The dressing percentage was calculated as HCW/(Final live BW × 0.96). The estimated empty body fat (EBF) percentage, final BW at 28% EBF (AFBW) [11], and the proportion of closely trimmed boneless retail cuts from the carcass round, loin, rib, and chuck (Retail Yield, RY; [12]) were calculated from observed carcass traits. Carcass data were available for all heifers except one from the RYE group.

### 2.4. Efficiency of Dietary NE Use Calculations

Observed dietary NE was calculated from the daily energy gain (EG) that was determined using the 1984 large-frame and compensating yearling heifer equation (EG; Mcal/d): EG = (Carcass-adjusted ADG from day 14 to 77)^1.119^ × 0.0608*W*^0.75^, where *W* is the mean BW (average BW using day 14 shrunk BW and FBW in kg; [13]). The maintenance energy required (EM; Mcal/d) was calculated by the following equation: EM = 0.077*W*^0.75^ [14]. Using the estimates required for maintenance and gain, the observed dietary net energy for maintenance (NEm) and net energy for gain (NEg) values [15] of the diet were generated using the following quadratic formula: $x=\;\frac{-b\pm \sqrt{b^{2}-4ac}}{2c}$, where *x* is the NEm (Mcal/kg), *a* = −0.41EM, *b* = 0.877EM + 0.41DMI + EG, *c* = −0.877DMI, and NEg was determined as 0.877NEm − 0.41 [16,17]. The observed-to-expected NE ratio was determined from the observed dietary NE for maintenance or gain divided by tabular NE for maintenance or gain.

### 2.5. Statistical Analysis

Growth performance, carcass traits, and efficiency of dietary NE use were analyzed as a randomized complete block design using the GLIMMIX procedure in SAS 9.4 (SAS Inst. Inc., Cary, NC, USA) with a pen as the experimental unit. The model included the fixed effect of dietary treatment, and a block (initial weight grouping) was included as a random variable. Least-squares means were generated using the LSMEANS statement of SAS, and treatment effects were analyzed using the PDIFF pairwise comparisons and the LINES option in SAS 9.4. The distribution of USDA yield and quality grade data was analyzed as binomial proportions in the GLIMMIX procedure of SAS 9.4 with fixed and random effects in the model, as described previously. An α-value of 0.05 or less determined significance, and tendencies were discussed between 0.05 and 0.10.

## 3. Results and Discussion

### 3.1. Cumulative Growth Performance (Day 1 to 77)

Growth performance responses are given in Table 2. There was no difference between treatments for the initial BW (*p* = 0.72). The final BW decreased by 6.8% in RYE compared to DRC heifers (*p* = 0.01); accordingly, the ADG decreased by 27.6% in RYE compared to DRC heifers (*p* = 0.01). It was demonstrated previously that complete replacement of dry-rolled corn with processed rye results in decreased growth performance in finishing steers [4]. The cumulative DMI tended to be greater by 6.4% in RYE compared to DRC heifers (*p* = 0.08); as such, feed efficiency decreased by 32.3% in RYE heifers compared to DRC heifers. Studies indicated that as higher amounts of processed rye are included in finishing diets, the DMI and gain efficiency linearly decrease [4].

### 3.2. Growth Performance in the Finishing Period (Day 14 to 77)

Data from the energetics assessment period are provided in Table 2. The BW on day 14 did not differ between treatments (*p* = 0.50). As previously mentioned, the final BW decreased by 6.8% in RYE heifers compared to DRC heifers. During the energetics assessment period, the ADG decreased by 29.0% (*p* = 0.01), and the DMI tended to be greater by 7.0% (*p* = 0.08) in RYE compared to DRC heifers. Complete replacement of dry-rolled corn with processed rye was shown to result in decreased intake and growth performance in finishing steers [4]. Alterations in daily gain and intake translated into reduced gain to a feed efficiency of 33.7% in RYE compared to DRC heifers (*p* = 0.01). Observed dietary NEm increased by 34.7% (*p* = 0.01) and observed dietary NEg increased by 46.8% in DRC compared to RYE heifers (*p* = 0.01). The observed-to-expected NE ratio for maintenance and gain also increased in DRC compared to RYE heifers (*p* ≤ 0.01). The observed-to-expected NEm ratio for the DRC diet was 1.14, far from the expected ratio of 1.00. Thus, applying substitution, the corresponding NEm value for dry-rolled corn is 2.73 Mcal/kg. This NE value is much greater than what current standards indicate [5], and if this value is used to estimate energy derivations by the replacement technique, it will result in aberrant values. The comparative energy value for unprocessed rye fed in the present study can be determined using the substitution technique to fit the NE value of the RYE diet, assuming that the NE content of the rest of the ingredients is constant, and only the NE of rye grain is adjusted to fit the observed diet NE. The NEg (Mcal/kg) value of the ingredient can be derived from NEm using the equation (NEg, Mcal/kg): 0.877NEm − 0.41 [16,17]. Accordingly, the NEm and NEg values for unprocessed rye are 1.73 and 1.11, Mcal/kg, respectively. Hence, based on growth performance, the NE value for unprocessed rye grain represents 78.6% of the energy value assigned by [5] for dry-rolled corn. This NE value of 1.73 Mcal/kg NEm is 9% lower than the NEm value reported by [4], who determined that the estimated NE value for rye grain processed to a processing index of 78.8% was 86% of the NE value for dry-rolled corn [4]. This indicates that regardless of the processing method, rye grain has less than 90% the NE value of dry-rolled corn. When comparing the results of the present study with those presented by [4], processing rye grain increases the NE value of rye by nearly 9%. This corresponds well to estimates for improvements in the NE value when small grains such barley or wheat are dry-rolled compared to when they are fed unprocessed [9,18,19,20].

### 3.3. Carcass Traits

Carcass trait responses are given in Table 3. DRC heifers showed a 7.5% increase in HCW compared to RYE heifers (*p* = 0.01). The dressing percentage was not influenced (*p* = 0.12) by dietary treatment. Reductions in HCW and dressing percentage were demonstrated when heifers were fed processed rye instead of dry-rolled corn [4]. The ribeye area and 12th rib fat thickness did not differ (*p* ≥ 0.14) due to dietary treatment. The marbling score tended to be greater by 12.7% in DRC heifers compared to RYE heifers (*p* = 0.10). The calculated yield grade increased by 9.6% and the retail yield decreased by 1.0% in DRC heifers compared to RYE heifers (*p* ≤ 0.01). The estimated EBF was greater by 5.1% in DRC compared to RYE heifers (*p* = 0.02), and the final BW at 28% EBF decreased by 12 kg in RYE heifers compared to DRC heifers (*p* = 0.01). There was no influence (*p* ≥ 0.13) of dietary treatment on the distribution of the USDA yield or quality grade. Studies have indicated that partial or complete replacement of dry-rolled corn with rye has a minimal influence on the distribution of the USDA yield or quality grade [4].

## 4. Conclusions

We conclude that unprocessed rye is a palatable feed ingredient for inclusion in finishing diets for beef cattle, as indicated by a greater DMI compared to dry-rolled corn, and it only minimally influences the carcass quality grade distribution when considering the reduced carcass fatness of heifers fed unprocessed rye. The feeding value of unprocessed rye is considerably less (21.4%) than that of dry-rolled corn and approximately 91% of the net energy value of processed rye. Hence, gain and gain efficiency are correspondingly lower when heifers are fed unprocessed rye as a replacement for dry-rolled corn in feedlot finishing diets fed to cattle.

## Figures and Tables

**Table 1:** Actual diet formulation and composition based upon weekly DM determinations and monthly ingredient composite nutrient compositions ^1^.

Item	Day 15 to 37		Day 38 to 59		Day 60 to 77	
	DRC	RYE	DRC	RYE	DRC	RYE
DRC ^2^, %	59.59	-	60.09	-	60.27	-
Unprocessed rye, %	-	59.68	-	60.00	-	60.18
CBCDS ^3^, %	20.17	20.12	19.80	19.84	-	-
DDGS ^4^, %	-	-	-	-	19.70	19.75
Grass hay, %	8.37	8.35	-	-	-	-
Oat hay, %	-	-	8.17	8.19	8.06	8.08
Meal supplement ^5^, %	7.00	6.98	7.03	7.05	7.03	7.04
Liquid supplement ^6^, %	4.87	4.86	4.92	4.93	4.95	4.95
Dry matter, %	75.02	75.97	74.32	74.92	87.62	88.51
Crude protein, %	12.86	15.79	12.97	15.71	13.69	16.43
NDF ^7^, %	21.14	27.09	19.65	25.68	20.18	26.22
ADF ^8^, %	9.84	13.41	8.78	12.40	9.25	12.87
Ash, %	6.84	7.16	6.72	7.03	6.04	6.35
EE ^9^, %	3.70	2.62	3.75	2.67	4.51	3.43
NEm ^10^, Mcal/kg	2.02	1.84	2.03	1.84	2.06	1.88
NEg ^11^, Mcal/kg	1.35	1.20	1.35	1.20	1.38	1.24

^1^ All values except for dry matter (DM) on a DM basis; ^2^ dry-rolled corn; ^3^ corn bran plus condensed distillers solubles; ^4^ dried distillers grains plus solubles; ^5^ contained (DM basis) 42.85% soybean hulls, 8.57% calcium carbonate, 48.58% ground corn, and melengestrol acetate (MGA; Zoetis, Parsippany, NJ) sufficient to provide 0.50 mg/heifer·d^−1^; ^6^ liquid supplement contained (DM basis) 43.26% crude protein, 38.83% nonprotein nitrogen, 0.95 Mcal/kg of net energy for maintenance, 0.64 Mcal/kg of net energy for gain, 1.07% ether extract, 13.18% total sugars, 54.02% ash, 11.02% calcium, 0.35% P, 7.08% K, 0.22% Mg, 5.05% NaCl, 2.93% Na, 0.39% S, 4.28 ppm Co, 202.18 ppm Cu, 12.13 ppm I, 6.92 mg/lb. of ethylenediamine dihydroiodide 113.72 ppm Fe, 308.33 ppm Mn, 2.93 ppm Se, 672.26 ppm Zn, 44,533 IU/kg of vitamin A, 445 IU/kg of vitamin E, and 649.4 g/Mg of monensin sodium (Rumensin, Elanco, Indianapolis, IN, USA); ^7^ neutral detergent fiber; ^8^ acid detergent fiber; ^9^ ether extract; ^10^ net energy for maintenance; ^11^ net energy for gain.

**Table 2:** Growth performance responses and efficiency of dietary net energy (NE) use.

Item	Dietary Treatment		Standard Error of the Mean	*p*-Value
	Dry-Rolled Corn (DRC)	Unprocessed Rye (RYE)		
Pens, *n*	4	4	-	-
Heifers, *n*	28	28	-	-
Cumulative day 1 to 77				
Initial body weight (BW) ^1^, kg	433	434	1.6	0.72
Final BW ^2^, kg	576	537	8.8	0.01
Average daily gain (ADG), kg	1.85	1.34	0.047	0.01
Dry matter intake (DMI), kg	11.52	12.26	0.277	0.08
ADG/DMI (G:F)	0.161	0.109	0.004	0.01
Finishing period (day 14 to 77)				
BW 14 ^1^, kg	449	447	3.2	0.5
Final BW ^2^, kg	576	537	8.8	0.01
ADG, kg	2	1.42	0.037	0.01
DMI, kg	12.35	13.22	0.339	0.08
G:F	0.163	0.108	0.0054	0.01
Observed dietary NE, Mcal/kg				
Maintenance	2.33	1.73	0.067	0.01
Gain	1.63	1.11	0.06	0.01
Observed-to-expected (O/E) NE ^3^				
O/E NEm	1.14	0.93	0.033	0.01
O/E NEg	1.2	0.91	0.041	0.01

^1^ The BW was shrunk by 4% to account for digestive tract fill; ^2^ hot carcass weight/0.625; ^3^ tabular NE (Mcal/kg) during the energetic assessment period for DRC was 2.04 and 1.36 for maintenance and gain, respectively and for RYE was 1.83 and 1.21 for maintenance and gain, respectively; tabular NEm and NEg for dry-rolled corn were assumed to be 2.20 Mcal/kg NEm and 1.50 Mcal/kg NEg; tabular NEm and NEg for unprocessed rye were assumed to be 1.90 Mcal/kg NEm and 1.26 Mcal/kg NEg.

**Table 3:** Carcass trait responses.

Item	Dietary Treatment		SEM	*p*-Value
	Dry-Rolled Corn (DRC)	Unprocessed Rye (RYE)		
Pens, *n*	4	4	-	-
Heifers, *n*	28	27	-	-
Hot carcass weight (HCW), kg	360	335	2.6	0.01
Dressing percentage ^1^, %	61.68	60.64	0.477	0.12
Rib fat, cm	1.32	1.19	0.069	0.14
Ribeye area cm^2^	87.4	85.79	1.535	0.37
Marbling ^2^	506	449	24.5	0.1
Yield grade	2.98	2.72	0.032	0.01
Retail yield ^3^, %	50.12	50.65	0.072	0.01
Estimated empty body fatness (EBF) ^4^, %	30	28.54	0.296	0.02
Final BW at 28% EBF (AFBW) ^4^, kg	535	523	1.4	0.01
Yield Grade distribution				
Y1, %	0	3.6	2.52	0.39
Y2, %	42.8	59.5	13.78	0.13
Y3, %	53.6	36.9	15.29	0.26
Y4, %	3.6	0	2.52	0.39
Quality Grade distribution				
Select, %	10.7	33.3	9.85	0.16
Choice,	35.7	40.5	12.82	0.81
Average choice, %	35.7	22	10.85	0.44
Top choice, %	10.7	4.2	5.66	0.47
Prime, %	7.1	0	5.05	0.39

^1^ HCW/final BW shrank by 4%; ^2^ USDA marbling score 400 = small^00^ = low choice; 500 = modest^00^ = average choice; ^3^ as a percentage of the HCW; ^4^ according to the equations described by Guiroy et al. (2002).

## Data Availability

Data available upon request.
